# Supplementary material for: A Pilot Study on the Effects of Transcranial Direct Current Stimulation on Brain Rhythms and Entropy during Self-Paced Finger Movement using the Epoc Helmet
Source: Front Hum Neurosci. 2017 Apr 25;11:201. doi: 10.3389/fnhum.2017.00201 (PMC5408787; doi:10.3389/fnhum.2017.00201)
Supplement: Supplementary file 1 [file Data_Sheet_1.zip › Complementary_results/CL-Ratio_average_PSD_windows/CL-Ratio_mean-win_F7-F8.pdf]

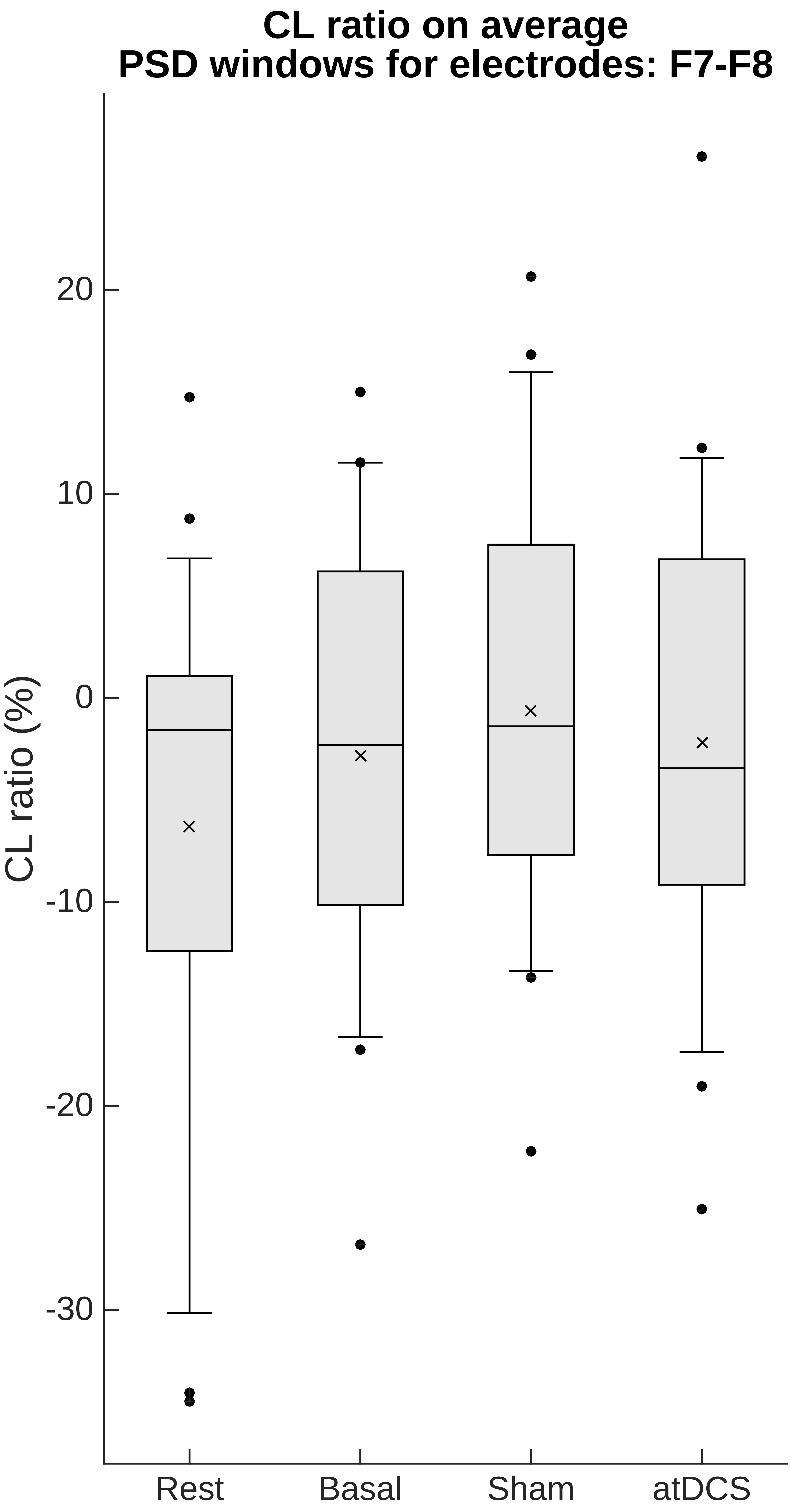
CL ratio on average
PSD windows for electrodes: F7-F8
CL ratio (%)
20
10
0
-10
-20
-30
Rest
Basal
Sham
atDCS
